# Assessment of Transverse Skeletal Differences Between Class I and Class II Malocclusion Using the J-J/Ag-Ag Ratio

**DOI:** 10.7759/cureus.108075

**Published:** 2026-04-30

**Authors:** Feras Alabrash, Fadi Khalil

**Affiliations:** 1 Dentistry - Department of Orthodontics, Latakia University, Lattakia, SYR

**Keywords:** cephalometric analysis, class ii malocclusion, class i malocclusion, craniofacial analysis, frontal cephalometry, j-j/ag-ag ratio, malocclusion, maxillary constriction, orthodontics, transverse discrepancy

## Abstract

Introduction: This study aimed to assess transverse skeletal differences between Class I and Class II malocclusion using the J-J/Ag-Ag ratio derived from frontal cephalometric analysis.

Methods: A retrospective cross-sectional study was conducted on 28 patients aged 18-30 years, equally divided into Class I and Class II malocclusion groups with balanced gender distribution. Frontal cephalometric radiographs were analyzed, and the J-J/Ag-Ag ratio was calculated for each subject. Statistical analysis included the Shapiro-Wilk test for normality and two-way analysis of variance (ANOVA) to evaluate the effects of malocclusion class and gender, with significance set at p < 0.05.

Results: A statistically significant difference in the J-J/Ag-Ag ratio was observed between Class I and Class II malocclusion groups (p < 0.001), with lower values recorded in Class II subjects, indicating relative transverse maxillary constriction. No significant effect of gender or interaction between class and gender was found. The effect size was large, confirming the clinical relevance of the findings.

Conclusion: The J-J/Ag-Ag ratio is a sensitive and reliable indicator for evaluating transverse skeletal discrepancies and can be effectively used in differentiating between Class I and Class II malocclusion in frontal cephalometric analysis.

## Introduction

Transverse skeletal discrepancies constitute a fundamental component in orthodontic diagnosis, particularly in patients with Class I and Class II malocclusions. Such discrepancies reflect disharmony between maxillary and mandibular transverse dimensions, which may adversely affect occlusal relationships, facial esthetics, and functional balance.

Accurate identification of these discrepancies is therefore essential for establishing a comprehensive diagnosis and guiding appropriate treatment strategies [1]. The transverse dimension plays a critical role in determining the underlying skeletal pattern and in planning orthodontic or orthopedic interventions. Failure to detect transverse deficiencies may lead to compromised treatment outcomes, occlusal instability, and an increased risk of relapse, highlighting the need for reliable and reproducible diagnostic methods [2].

CBCT-based randomized controlled trials have demonstrated significant skeletal transverse changes following maxillary expansion, underscoring the clinical importance of accurate transverse assessment [3]. Conventionally, transverse relationships have been assessed through clinical examination, dental cast analysis, and radiographic techniques. Among these, cephalometric analysis, particularly posteroanterior cephalometry, remains a widely used method for evaluating craniofacial morphology and intermaxillary relationships in orthodontic practice [4].

However, conventional cephalometric assessment is associated with several limitations, including landmark identification errors, operator-dependent variability, and inconsistencies in measurement reproducibility. Additionally, the inherent limitations of two-dimensional imaging in representing three-dimensional structures may reduce diagnostic accuracy [2,5,6]. In contrast, cone-beam computed tomography (CBCT) provides a three-dimensional assessment of craniofacial structures, allowing more accurate evaluation of transverse skeletal relationships and overcoming the inherent limitations of two-dimensional imaging [7].

To overcome these limitations, proportional indices have been proposed as more reliable indicators of transverse skeletal relationships. In this context, the J-J/Ag-Ag ratio has been introduced as a quantitative cephalometric parameter that reflects the relationship between maxillary width and mandibular basal width. This ratio provides a simplified and reproducible method for assessing transverse discrepancies using frontal cephalometric radiographs [8,9].

Despite its potential clinical relevance, the current literature lacks sufficient evidence regarding the standardized application of the J-J/Ag-Ag ratio in comparative analyses. In particular, there is a paucity of studies investigating transverse skeletal differences between Class I and Class II malocclusions using this parameter, which represents a notable gap in orthodontic research [2,10]. However, CBCT-based studies have provided additional insights into mandibular dental and basal arch dimensions in these groups [11], which support the need for further comparative investigation.

Therefore, this retrospective radiological study aimed to assess and compare transverse skeletal discrepancies between Class I and Class II malocclusions using the J-J/Ag-Ag ratio as a quantitative diagnostic indicator. The J-J/Ag-Ag ratio is defined as the ratio between maxillary basal width (J-J) and mandibular basal width (Ag-Ag), reflecting transverse maxillomandibular proportionality. Values close to 0.80 are generally considered indicative of transverse harmony, whereas lower values may suggest maxillary constriction [12].

It was hypothesized that significant differences in the J-J/Ag-Ag ratio would be observed between Class I and Class II malocclusions, reflecting variations in transverse maxillomandibular relationships.

## Materials and methods

Study design

This retrospective radiological study was conducted to evaluate transverse skeletal relationships in subjects with Class I and Class II malocclusions using the J-J/Ag-Ag ratio derived from posteroanterior (PA) cephalometric analysis.

Sample selection

A total of 28 PA cephalometric radiographs were included and divided into two groups: 14 subjects with skeletal Class I malocclusion and 14 subjects with skeletal Class II malocclusion. Each group consisted of seven males and seven females, aged between 21 and 30 years. The radiographs were obtained from patients attending the Department of Orthodontics at Latakia University and private orthodontic clinics.

Inclusion and exclusion criteria

The inclusion and exclusion criteria are summarized in Table 1.

**Table 1:** Inclusion and exclusion criteria PA: Posteroanterior.

Inclusion Criteria	Exclusion Criteria
Adult patients aged 21-30 years	Previous orthodontic treatment
Skeletal Class I or Class II malocclusion	History of orthognathic surgery
Complete permanent dentition (excluding third molars)	Craniofacial anomalies or syndromes
High-quality standardized PA cephalometric radiographs	Poor-quality radiographs

Data collection and classification

All radiographs were obtained using a standardized imaging protocol with the same radiographic device (Vatech Pax-I, 2023; Vatech Co., Ltd., Korea). Skeletal classification was determined based on the ANB (A point-Nasion-B point) angle obtained from lateral cephalometric analysis, where Class I was defined as ANB between 0° and 4° and Class II as ANB greater than 4° [13].

All radiographs were obtained under standardized conditions with consistent magnification, and no additional scaling adjustments were required during analysis.

Image processing and measurements

PA cephalometric radiographs were analyzed using WebCeph software (AssembleCircle, Korea). Landmark identification was initially performed using artificial intelligence-assisted detection, followed by manual verification and adjustment by the examiner to ensure accuracy.

**Figure 1:**
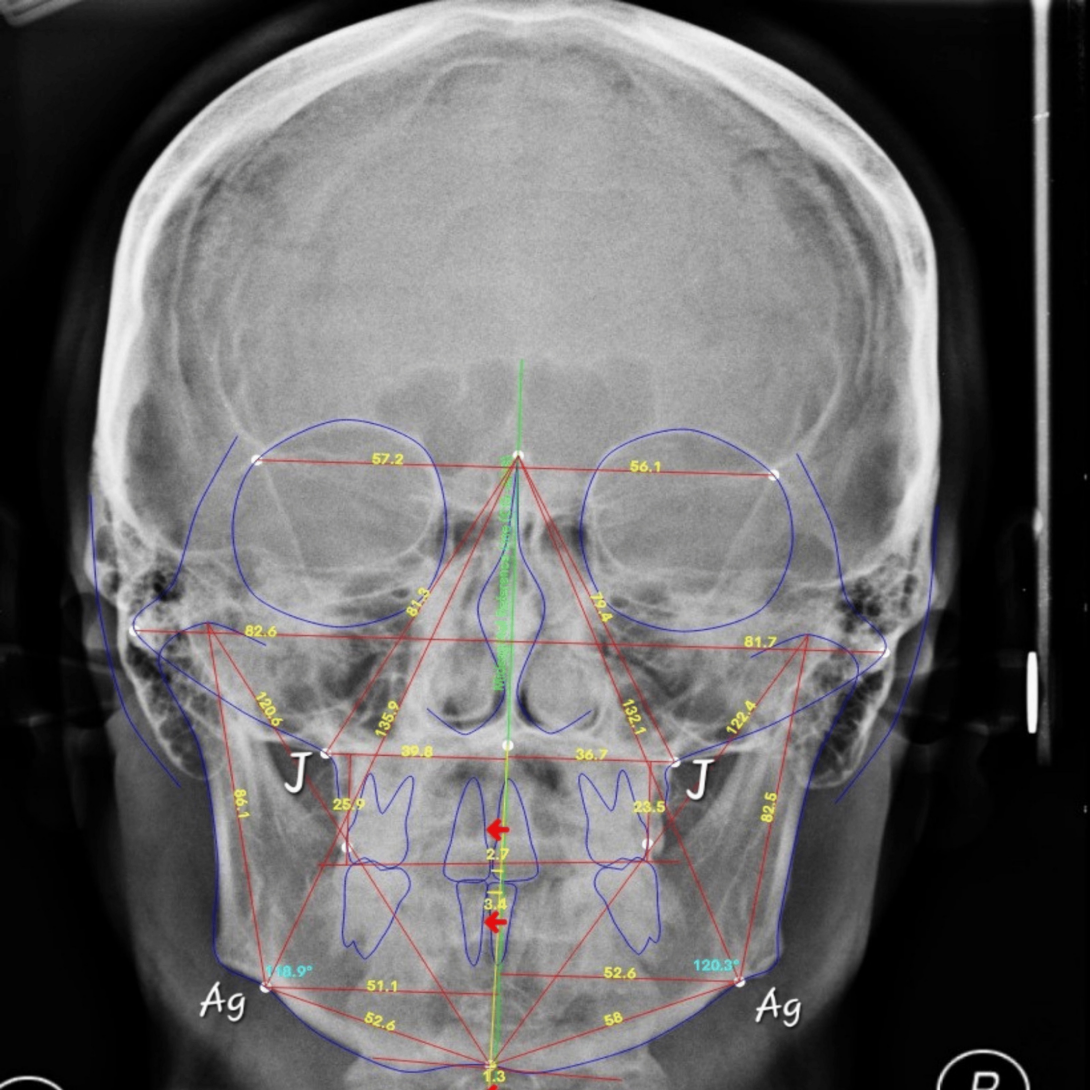
Posteroanterior cephalometric radiograph illustrating the measurement of the J-J/Ag-Ag ratio. The interjugale distance (J-J) and interantegonial distance (Ag-Ag) are indicated, representing the transverse dimensions of the maxilla and mandible. Source: Authors of this study.

The J point was defined as the most lateral point on the maxillary alveolar process, while Ag (antegonial notch) was defined as the most inferior point at the antegonial notch of the mandible on each side. The transverse skeletal relationship was assessed using the J-J/Ag-Ag ratio, calculated by dividing the interjugale distance (J-J) by the interantegonial distance (Ag-Ag). The method of measurement is illustrated in Figure 1.

This ratio reflects the proportional transverse relationship between the maxillary and mandibular skeletal bases. A value approaching 0.80 has been suggested as indicative of transverse skeletal harmony, whereas deviations from this threshold may indicate maxillomandibular transverse discrepancies [12].

Measurement reliability

To assess measurement reliability, 10 radiographs were randomly selected and re-measured after a two-week interval by the same examiner. Intra-examiner reliability was evaluated using the intraclass correlation coefficient (ICC), demonstrating high reproducibility (0.87).

Statistical analysis

Statistical analysis was performed using SPSS software (IBM Corp., Armonk, NY). Descriptive statistics were calculated for all variables. Data normality was assessed using the Shapiro-Wilk test. Logarithmic transformation was applied when required. A two-way analysis of variance (ANOVA) was used to evaluate the effects of skeletal class and sex on the J-J/Ag ratio, including their interaction. Effect size was assessed using partial eta squared (η²p), and statistical significance was set at p < 0.05.

## Results

A total of 28 subjects were included in this study, equally distributed between Class I and Class II malocclusion groups, with equal representation of males and females.

Descriptive statistics showed that the mean J-J/Ag-Ag ratio was higher in Class I subjects (mean = 76.79, SD = 0.90) compared to Class II subjects (mean = 71.35, SD = 2.04), as illustrated in Table 2.

**Table 2:** Descriptive statistics of J-J/Ag-Ag ratio

Group	Mean	SD
Class I	76.79	0.90
Class II	71.35	2.04

Two-way ANOVA revealed a statistically significant effect of skeletal class on the J-J/Ag-Ag ratio (F = 77.36, p < 0.001, η² = 0.763). In contrast, no statistically significant effect of sex was observed (F = 0.043, p = 0.837, η² = 0.0004). Similarly, the interaction between skeletal class and sex was not statistically significant (F = 0.034, p = 0.855, η² = 0.0003).

Post hoc power analysis demonstrated a statistical power of 1.0, indicating that the sample size was sufficient to detect the observed differences. As illustrated in Table 3, a clear difference in the J-J/Ag-Ag ratio between Class I and Class II groups was observed.

**Table 3:** Two-way ANOVA results for J-J/Ag-Ag ratio ANOVA: Analysis of variance.

Source	F	p-value	Partial η²
Class	77.36	<0.001	0.763
Gender	0.043	0.837	0.0004
Class × Gender	0.034	0.855	0.0003

## Discussion

The present study demonstrated a statistically significant reduction in the J-J/Ag-Ag ratio in Class II subjects compared to Class I, indicating a substantial alteration in transverse maxillomandibular proportionality. This finding reflects a relative imbalance between maxillary and mandibular basal widths in Class II malocclusion.

Measurement reliability was assessed using the ICC, demonstrating high intra-examiner consistency (0.87). However, inter-examiner reliability was not evaluated, which may limit the generalizability of the measurements.

This result can be interpreted within the framework of craniofacial morphology, as skeletal Class II individuals have been shown to present maxillary transverse deficiency and altered transverse relationships [14,15]. A decrease in the J-J/Ag-Ag ratio may therefore reflect maxillary constriction, increased mandibular width, or a combination of both. Although PA cephalometry remains a widely used method for transverse assessment, it is inherently limited by its two-dimensional nature, which may lead to projection errors, superimposition of anatomical structures, and reduced accuracy in landmark identification. In contrast, CBCT provides three-dimensional visualization, allowing more precise assessment of craniofacial structures and transverse relationships. However, despite its superior diagnostic accuracy, CBCT use should be justified due to higher radiation exposure and cost. Previous studies have demonstrated the greater diagnostic accuracy of CBCT compared with conventional radiography in craniofacial assessment [16], supporting the interpretation of the present findings while acknowledging the limitations of two-dimensional analysis.

The present findings are consistent with CBCT-based studies demonstrating transverse discrepancies associated with skeletal Class II malocclusion. Hajeer et al. and Sánchez-Morillo et al. [17,18] reported a significant association between maxillary transverse deficiency and skeletal Class II patterns. Similarly, Ye** **et al. [20] demonstrated that transverse width indices are effective diagnostic tools for identifying maxillary transverse deficiency, supporting the clinical relevance of proportional assessment.

Furthermore, Lee et al. [15] provided CBCT-based normative data for transverse dimensions in individuals with normal occlusion, which may serve as a reference for identifying deviations in malocclusion cases. The reduced J-J/Ag-Ag ratio observed in Class II subjects in the present study may therefore reflect deviation from normal transverse relationships. These findings indicate an association between sagittal skeletal pattern and transverse proportional relationships, rather than a direct causal relationship.

In contrast, transverse discrepancies may not be exclusively determined by sagittal skeletal classification but may also be influenced by variability in measurement techniques and anatomical structures. Kong et al.[14] demonstrated that different transverse measurement methods may yield variable diagnostic outcomes, which may explain inconsistencies among studies.

From a methodological perspective, proportional indices such as the J-J/Ag-Ag ratio may provide a normalized assessment of intermaxillary relationships by reducing the influence of overall craniofacial size. This concept is supported by studies emphasizing the importance of appropriate diagnostic indices in transverse evaluation [19,20].

Clinically, the identification of transverse discrepancies in Class II patients is relevant for treatment planning, particularly in cases requiring maxillary expansion, as emphasized in recent diagnostic studies [19]. Previous studies have also suggested a relationship between skeletal transverse discrepancies and functional adaptations, including alterations in masticatory muscle activity [21].

No statistically significant effect of sex on the J-J/Ag-Ag ratio was observed. This may suggest relative stability of transverse proportional relationships between males and females; however, further studies specifically addressing sex-related differences are required.

The J-J/Ag-Ag ratio may serve as a useful screening indicator for transverse discrepancies; however, its clinical application should be interpreted with caution.

The strengths of this study include the use of a proportional index and a standardized measurement protocol. However, several limitations should be acknowledged. First, the retrospective design and relatively small sample size may limit the generalizability of the findings. Second, the use of a convenience sampling method based on available radiographs may introduce selection bias. Third, although measurement reliability was high, only intra-examiner reliability was assessed. Additionally, the very small standard deviation observed in the Class I group may reflect limited variability within the sample or potential underestimation of variance. Finally, the use of two-dimensional PA cephalometry may introduce projection errors and distortions compared with three-dimensional CBCT imaging, which should be considered when interpreting the results.

Additionally, two-dimensional cephalometric analysis may not fully represent three-dimensional transverse relationships, whereas CBCT-based methods provide a more comprehensive evaluation [20]. Future research should focus on validating proportional indices such as the J-J/Ag-Ag ratio in larger populations and integrating them with CBCT-based approaches to enhance diagnostic accuracy.

## Conclusions

The J-J/Ag-Ag ratio showed a significant reduction in Class II malocclusion compared to Class I, reflecting altered transverse maxillomandibular proportions. This parameter may serve as a useful indicator of transverse skeletal relationships and may aid in the assessment of transverse discrepancies in orthodontic evaluation. However, these findings should be interpreted with caution due to the retrospective design and limited sample size. Further studies with larger, prospectively collected samples and CBCT-based validation are recommended.
